# ATF4 activity: a common feature shared by many kinds of slow-aging mice

**DOI:** 10.1111/acel.12264

**Published:** 2014-08-26

**Authors:** Weiquan Li, Xinna Li, Richard A Miller

**Affiliations:** 1 Department of Pathology, University of MichiganAnn Arbor, MI, 48109, USA; 2 Geriatrics Center, University of MichiganAnn Arbor, MI, 48109, USA

**Keywords:** acarbose, caloric restriction, longevity, methionine restriction, rapamycin

## Abstract

ATF4, a DNA-binding factor that modulates responses to amino acid availability and ribosomal function, has been shown to be altered in both liver and fibroblasts from two strains of long-lived mice, i.e. Snell dwarf and PAPP-A knockout mice. New data now show elevated ATF4 levels, and elevation of ATF4-dependent proteins and mRNAs, in liver of mice treated with acarbose or rapamycin, calorically restricted mice, methionine-restricted mice, and mice subjected to litter crowding. Elevation of ATF4, at least in liver, thus seems to be a shared feature of diets, drugs, genes, and developmental alterations that extend maximum lifespan in mice.

## Introduction

ATF4 is a transcriptional factor which senses deficits in protein translation, typically related to ER stress or amino acid limitation, and in turn activates a group of target genes through a consensus ATF4 promoter binding motif (Harding *et al*., 2003; Su *et al*., 2009). A role for ATF4 in modulation of aging was suggested by evidence that lifespan extension in yeast, whether caused by 60S ribosome mutations, lower TOR function, or nutrient deprivation, depended on the yeast homologue, Gcn4 (Steffen *et al*., 2008).

To see whether ATF4 might contribute to extended longevity in Snell dwarf mice, we evaluated ATF4 status in fibroblasts and liver of young adult Snell dwarf mice. In Snell dwarf fibroblasts, we noted augmented ATF4 responses to amino acid withdrawal, cadmium, H_2_O_2_, and the ER stress agent tunicamycin, with parallel increases in ATF4 itself as well as in multiple ATF4 target genes (Li & Miller, 2014). Liver of Snell dwarf mice also showed elevation of ATF4 protein, and of mRNA and protein for multiple ATF4 target genes. Parallel findings were also noted in fibroblasts and liver of PAPP-A KO mice, in which lifespan extension (Conover & Bale, 2007) is thought to reflect diminished IGF-1 availability secondary to higher tissue levels of IGF-1 binding proteins. Thus, both of these long-lived mice, with alterations in GH and/or IGF-1 action, showed augmented ATF4 levels in liver and heightened ATF4 responsiveness in skin-derived fibroblasts subjected to oxidant or ER stress.

To see whether elevated ATF4 function might be a feature shared by genetic and nongenetic models of slow aging in mice, we have now evaluated ATF4, ATF4-controlled proteins, and ATF4-controlled mRNA levels in liver of five kinds of mice known to have increased maximal longevity caused by nongenetic interventions: mice treated with acarbose (Harrison *et al*., 2014) or rapamycin (Harrison *et al*., 2009; Miller *et al*., 2011), mice subjected to caloric restriction (Flurkey *et al*., 2010) or methionine restriction (Miller *et al*., 2005; Sun *et al*., 2009), and mice kept in crowded litters from birth to weaning (Sun *et al*., 2009).

Acarbose (Aca) inhibits α-glucosidases in the intestine, thereby slowing the breakdown of complex polysaccharides to sugars and thus blunting the spike in blood glucose levels that follows a meal. It is an FDA-approved agent for treatment of human diabetes. Food intake is typically increased in Aca-treated mice (Yamamoto & Otsuki, 2006). The NIA Interventions Testing Program (ITP) has found (Harrison *et al*., 2014) that Aca increases median lifespan in genetically heterogeneous UM-HET3 mice by 22% in males (*P *< 0.0001) and by 5% in females (*P *= 0.01). Body weight was reduced, at 12 months of age, by 14% in males and by 22% in females, suggesting that the sexual dimorphism in longevity response does not parallel effects on body weight. Aca lowers fasting insulin levels in males but not in females, lowers IGF-1 levels in both sexes, and leads to a significant increase in blood levels of FGF-21, a mediator that declines dramatically in mice on a calorie-restricted (CR) diet (Harrison *et al*., 2014). Maximum lifespan, or more specifically the likelihood of survival to the 90th percentile age of the joint survival distribution (Wang *et al*., 2004), is increased in both males and females (*P *< 0.0001).

Rapamycin, an inhibitor of mTOR kinase, increases lifespan of both male and female UM-HET3 mice whether started at 9 or at 20 months of age (Harrison *et al*., 2009; Miller *et al*., 2011, 2014). At a dose of 42 parts per million (ppm), rapamycin leads to an increase of 23% in male lifespan and 26% in female lifespan (Miller *et al*., 2014). The stronger percentage response in females may reflect higher blood levels in females, compared with males consuming the same proportion of rapamycin in food (Miller *et al*., 2014). Rapamycin-treated mice also show retardation of age-related deficits in liver, tendon, heart, endometrium, and adrenal tissues, and delay in the age-sensitive decline in spontaneous activity (Wilkinson *et al*., 2012). Some age-sensitive physiological endpoints show improvement shortly after rapamycin treatment, some show beneficial effects only at older ages, and some do not show any improvement in an analysis of male C57BL/6 mice (Neff *et al*., 2013). Rapamycin also retards or reverses age-related declines in bone marrow production of B lymphocytes (Chen *et al*., 2009), age-dependent deficits in pulmonary responses to bacterial infection (Hinojosa *et al*., 2012), and development of both pathologic changes and behavioral deficits in a mouse model of Alzheimer's disease (Caccamo *et al*., 2010). Rapamycin impairs glucose handling in multiple ways, with deficits in insulin production and response contributing in ways that depend on dose and time of exposure.

Caloric restriction (CR) is the best-studied method for slowing aging and extending lifespan in rodents, with beneficial effects on dozens of strains of rats and mice (Weindruch & Walford, 1988), including the UM-HET3 mice used in this study and the ITP protocol (Flurkey *et al*., 2010). Because CR diets diminish mTOR function, it has been suggested that inhibition of mTOR may be a shared feature of the anti-aging pathways stimulated by CR and rapamycin (Johnson *et al*., 2013), but UM-HET3 mice exposed to rapamycin or to CR diets differ in many ways, including effects on fasting insulin and glucose levels, alteration in serum FGF-21 levels (depressed by CR diet and increased by rapamycin), and expression of hepatic genes involved in metabolism of xenobiotics (Miller *et al*., 2014).

Diets with reduced levels of methionine (‘Meth-R’ diets) lead to lifespan extension of 40% or more in rats and extend lifespan of multiple strains of rats despite an increase in food consumed per gram of body mass (Zimmerman *et al*., 2003). Meth-R diet also increases maximum lifespan in CB6F1 mice whether initiated at 6 weeks or at 12 months of age and retards development of age-related declines in T cell subset distribution and eye lens transparency (Miller *et al*., 2005; Sun *et al*., 2009). Although Meth-R mice weigh less than controls and have lower levels of IGF-1, insulin, and glucose (Miller *et al*., 2005), there are many physiological differences between the CR and Meth-R systems. There is, for example, little overlap in patterns of changed hepatic gene expression, and Meth-R mice do not show the increase in p38 and pERK kinase activation and decline in mTOR function seen in liver of CR mice (Sun *et al*., 2009).

In the crowded litter (CL) model, mice are foster nursed in groups of 12 (‘CL12’) pups per mother and compared with controls suckled in groups of 8 per mother (CL8 controls). Pups are weaned at 3 weeks of age and given unrestricted access to food thereafter. CL12 mice live 18% longer than controls (*P *= 0.0007) and show a significant increase in maximum lifespan (Sun *et al*., 2009). Although the intervention is thus limited to the first three weeks of life, patterns of liver gene expression remain distinct from controls at both 12 and 22 months of age (Steinbaugh *et al*., 2012), and CL12 and CL15 mice show alterations in glucose tolerance and leptin sensitivity that last at least to 22 months (Sadagurski *et al*., 2014).

## Results

### Increased expression of ATF4, ATF3, and CHOP proteins in liver of five kinds of slow-aging mice

**Figure 1:**
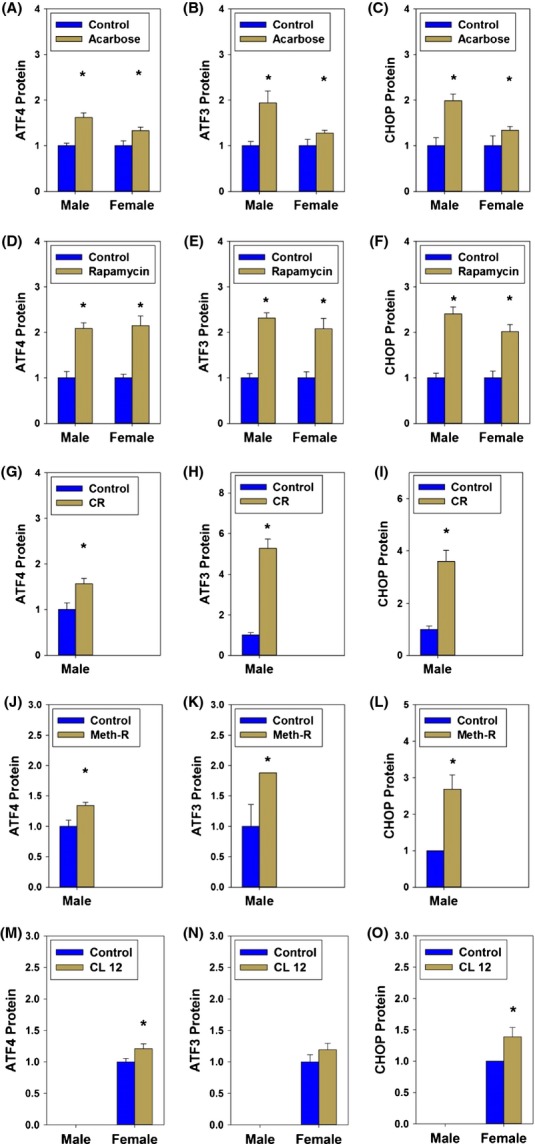
Quantitation of protein expression of ATF4 and ATF4 targets. Levels of ATF4 protein (left: A, D, G, J, and M), ATF3 protein (middle: B, E, H, K, and N) and CHOP protein (right: C, F, I, L, and O) in liver of mice exposed to acarbose (top row: A, B, and C), rapamycin (second row: D, E, and F), calorie restriction (third row: G, H, and I), methionine restriction (fourth row: J, K, and L), or crowded litter intervention (bottom row: M, N, and O). Bars show means and SEM for N = 6 mice per age/condition shown. Only male mice were available for testing in the CR and Meth-R condition, and only female mice in the CL condition. Asterisk (*) indicates a significant effect of the intervention, at *P *< 0.05, using Sidak's post hoc test after a one-factor analysis of variation.

**Figure 2:**
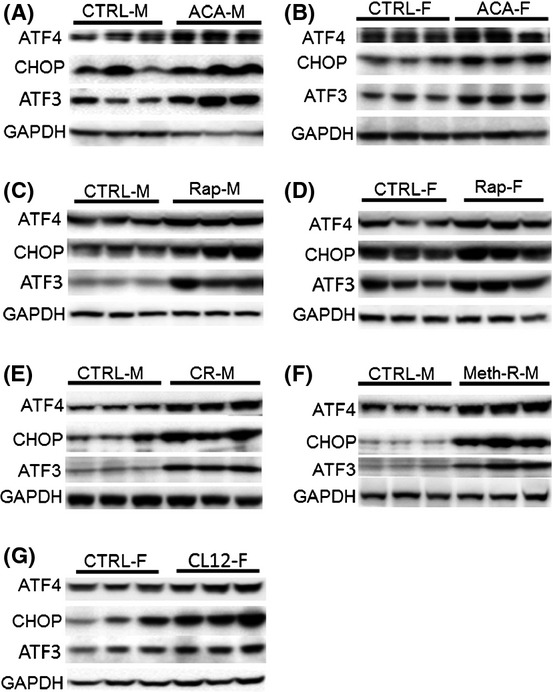
Representative images showing protein expression of ATF4 and ATF4 targets. Protein expression of ATF4 and ATF4 targets by Western blot in livers of mice exposed to acarbose (A: male; B: female), rapamycin (C: male; D: female), calorie restriction (E: male), methionine restriction (F:male), or crowded litter intervention (G:female).

Both male and female mice exposed to acarbose from 4 to 12 months of age had significant elevations of ATF4 protein in liver (Fig.1A). ATF3 and CHOP proteins, which are known to be modulated by ATF4 function, were also higher in acarbose-treated male and female mice (Fig.1B,C). Figure2A and B show representative immunoblots for these Aca-treated mice and controls. Similarly, ATF4, ATF3, and CHOP protein levels were elevated in male and female rapamycin-treated mice (age 22 months; Figure 1DEF; see Figure 2CD for representative immunoblot images). Significant increases in all three proteins were also seen in male mice from a group of CR mice (calorie restricted from 1.5 to 12 months; Figs1G–I and 2E), and in 18-month-old male mice from a methionine-restricted cohort (Figs1J–L and 2F). Lastly, female mice from a CL cohort, evaluated at 6 months, were found to have higher levels of all three proteins, with a significant effect (*P *< 0.05) seen for ATF4 and CHOP but not for ATF3 (Figs1M–O and 2G).

### Increased transcription of ATF4 target genes in five slow-aging mouse models

**Figure 3:**
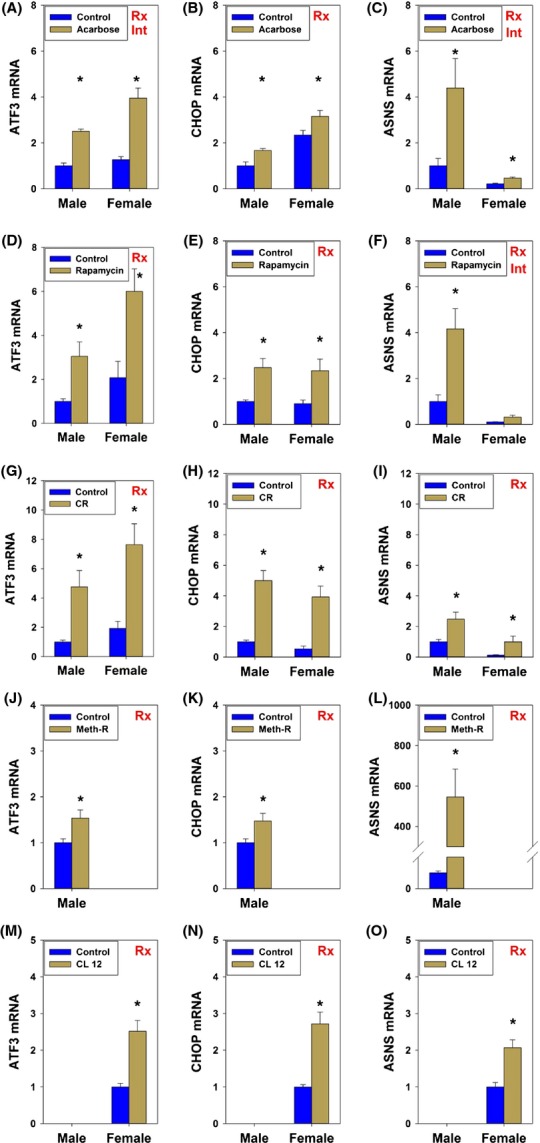
Transcription of ATF4 targets in liver of long-lived mice. Levels of mRNA for ATF3 (left), CHOP (middle), and ASNS protein (right) in liver of mice exposed to acarbose (top row: A, B, and C), rapamycin (second row: D, E, and F), calorie restriction (third row: G, H, and I), methionine restriction (fourth row: J, K, and L), or crowded litter intervention (bottom row: M, N, and O). Bars show means and SEM for N = 6 mice per age/condition shown. Only male mice were available for testing in the CR and Meth-R condition, and only female mice in the CL condition. Data were analyzed by two-factor ANOVA, with tests for effects of intervention, sex, and the [sex × intervention] interaction term. Panels with the ‘Rx’ indicator had a significant overall effect of the intervention, and those with the ‘Int’ indicator had a difference between males and females in the response to the intervention. Asterisk (*) indicates a significant effect of the intervention, at *P *< 0.05, using Sidak's *post hoc* test after a one-factor analysis of variation.

ATF4 regulates transcription of many genes, including (in liver) ATF3 (activating transcription factor 3), CHOP (CCAAT/enhancer-binding protein homologous protein, also called GADD15), and ASNS (asparagine synthetase). Figure3 shows hepatic mRNA levels for these three genes in each of the five varieties of slow-aging mice. When both sexes were available for study (acarbose, rapamycin, and CR), a two-factor analysis of variation was used to test for the overall effect of the intervention on the mRNA and to evaluate the interaction term as an indication of possible sex effects in responses to the intervention. The top line of Fig.3 (panels ABC) shows that acarbose leads to a significant increase in ATF3, CHOP, and ASNS mRNA in liver of both male and female mice. The ‘Rx’ shown in each panel, derived from the two-factor ANOVA, indicates that the overall effect of acarbose is statistically significant, and the ‘Int’ designation, for ATF3 and ASNS, indicates that the two sexes had significantly different responses: ATF3 response was higher in females, and ASNS response was higher in males.

Figure3 also shows significant overall effects of rapamycin and CR diet on each of the three ATF4-modulated mRNAs. The effects are significant for each sex considered independently, except for ASNS in female mice of the rapamycin cohort; the ANOVA showed that male and female responses to ASNS to rapamycin were indeed significantly different. Male mice treated with a methionine-restricted diet (Fig.3, panels JKL) showed significant increases in each of the three mRNAs (females were not available for analysis). Methionine restriction produced a very large increase, over 400-fold, in ASNS mRNA, suggesting that this mRNA may be regulated by amino acid restriction in ways that do not reflect merely altered ATF4 function. For the CL intervention, only females were available for analysis, and these showed a significant increase in each of the three mRNA tested.

As a control, we examined expression of a panel of liver genes whose expression level is not thought to be related to ATF4 action, including Bnip, ATG12, NRF2, PEPCK1, Sirt1, and FasL, in each of the five varieties of slow-aging mice, and found no significant differences between treated and control mice (data not shown) for any of these mRNAs.

We also measured ATF4 mRNA in liver of each of the five varieties of slow-aging mice. Acarbose treatment led to a significant increase in ATF4 mRNA in males and females. Rapamycin treatment led to an increase in ATF4 mRNA in both sexes, significant in males. Methionine-restricted males showed a significant increase (females were not available for study). CR diet led to an increase in ATF4 mRNA in both sexes, but the effect was not statistically significant. Female CL mice showed a significant increase in ATF4 mRNA (males were not available for study). Thus, ATF4 mRNA levels in liver were elevated in all eight comparisons, with significant effects in 5 of the 8. The data are thus consistent with the protein measurements shown in Fig.1.

To see whether ATF4-regulated mRNAs were modulated in other tissues, we measured the same three mRNA species in kidney and heart of mice exposed to acarbose, rapamycin, or CR diet, using six males and six females of each treated and age-matched control group (data not shown). All three mRNAs were significantly increased in kidney of male and female CR mice, and ATF3 mRNA increased significantly in kidney of rapamycin treated males and females. For heart, we saw significant increases in ATF3 in male and female CR mice and of ASNS in male and female rapamycin-treated mice. We did not see any significant change in heart or kidney of acarbose-treated mice. Thus, our data show that ATF4 elevation is characteristic of liver in all five of the tested slow-aging mice (plus Snell and PAPP-A KO mice, reported previously), but effects of acarbose, rapamycin, and CR on kidney and heart are not as consistent as in liver.

## Discussion

The availability of multiple methods to extend mouse maximal lifespan – genetic, dietary, development, or drug-induced – provides an opportunity to test the hypothesis that augmented ATF4 action, necessary for multiple modes of lifespan extension in yeast (Steffen *et al*., 2008), is also characteristic of slow aging in mice. Data in this paper show that ATF4 levels, levels of proteins controlled by ATF4, and levels of three mRNAs regulated directly by ATF4 are elevated in liver of mice exposed to each of five interventions shown elsewhere to increase maximal longevity: the drugs acarbose and rapamycin, diets low in calories or methionine, or transient milk deprivation limited to the suckling period. Our previous work (Li & Miller, 2014) has shown similar increases in ATF4 protein and downstream indicators of ATF4 function in liver of Snell dwarf mice and PAPP-A knock-out mice, mutations that increase maximal lifespan and health in old age by alteration of endocrine pathways connected to GH and/or IGF-1. The previous study also documented augmented ATF4 responses in fibroblast cell lines derived from skin of adult Snell and PAPP-A KO mice, suggesting that the relevant changes affect more than a single cell type and that the changes include epigenetic modifications preserved during multiple mitotic cycles in tissue culture medium. All of these data are consistent with the idea that elevation of ATF4 function may contribute to the slow aging and extended lifespan in each of these diverse varieties of mice.

Levels of the mRNA for ATF4 itself were augmented in each of the 8 combinations of sex and intervention and reached statistical significance for 5 of the 8 comparisons. Although ATF4 activity is largely controlled by post-transcriptional changes, including proteasomal degradation, ubiquitination, phosphorylation, and heterodimerization with co-factors, our results suggest that alteration or production of stability of ATF4 mRNA may also contribute to the increase in ATF4 protein and activity seen in each of these five kinds of slow-aging mice.

We chose to study ATF3, CHOP, and ASNS as representatives of the much larger set of mRNAs whose transcription is sensitive to ATF4 activity, but it would be incorrect to assume that these mRNA levels are regulated by ATF4 alone. The increases in mRNA and protein levels for the tested genes are not in all cases closely paralleled by the degree of increase in ATF4 protein in the same tissue. This is not surprising, as genes whose transcription is modulated by ATF4 are also susceptible to the influence of other regulatory pathways. Changes in ASNS mRNA, for example, are in some of our models disproportional to the change in ATF4 protein and to changes in ATF3 and CHOP mRNAs. We note, however, that ASNS mRNA levels can be altered by other mechanisms, including some related to ATF5 (Al Sarraj *et al*., 2005; Zhou *et al*., 2008) and others dependent on DNA methylation of the ASNS promoter region (Sugiyama *et al*., 1983; Ren *et al*., 2004) Thus, we do not see the disparity among protein levels of ATF4-regulated genes, in different kinds of slow-aging mice, as inconsistent with our central conclusion that increased ATF4 action is a shared characteristic of seven kinds of slow-aging mice, including the five models for which data are presented in this study.

While we have not undertaken a comprehensive analysis of the effects of age, sex, and background stock on ATF4, we do note that elevation of liver ATF4 function is seen in male and female mice, at ages from 6 to 22 months, and on at least four genetic backgrounds (CB6F1 and UM-HET3 in the current study, and segregating stocks for the Snell and PAPP-A KO mice in the earlier report). We note that mRNA for two of the ATF4-sensitive genes shows a sexually dimorphic response to acarbose, with females responding more dramatically for ATF3 expression and less strongly than males with respect to ASNS. Acarbose leads to significant lifespan extension in both males and females, but a much greater response in the males (22% increase, compared with 5% in females) (Harrison *et al*., 2014). A much wider survey of ATF4-responsive mRNAs would be needed to develop hypotheses about patterns of sex-specific modulation of responses to acarbose that might contribute to the sex-specificity of the lifespan results.

Our data make a testable prediction: drugs or diets or other interventions that increase maximal lifespan in mice should lead to elevation of ATF4, at least in liver and perhaps in other tissues. Evidence in support of this idea would provide further justification for analysis of the role of ATF4 in postponement of aging, while discovery of interventions that slow aging without modulation of ATF4 are also likely to be of great interest.

Our approach does not provide definitive evidence that higher ATF4 function is either necessary or sufficient for the extended longevity and extended good health seen in these many kinds of slow-aging mice. The data do, however, provide strong grounds for testing such ideas in future studies. For example, elevation of ATF4 might be used as a screen, in cell lines or in genetically malleable invertebrate organisms, for drugs that might deserve testing for beneficial effects in mice. Evaluation of ATF4 pathways in multiple tissues might help to reveal tissues in which altered ATF4 action is typically associated with slow aging. Our data, in conjunction with the data on yeast which inspired our study (Steffen *et al*., 2008), provide justification for evaluation of ATF4 dependent pathways in multiple vertebrate and invertebrate organisms.

Studies of ATF4-null mice have documented roles for ATF4-dependent pathways at several developmental stages and in adult life. ATF4-deficient mice show defects in eye development (Tanaka *et al*., 1998) and in production of erythrocytes by the fetal liver (Masuoka & Townes, 2002), as well as bone formation (Yoshizawa *et al*., 2009), angiogenesis (Wang *et al*., 2013), and elements of learning and memory (Costa-Mattioli *et al*., 2007). Muscle-specific ATF4 knockout mice show reduced induction of Gadd45a mRNA in response to stress and as a result show less muscle atrophy after fasting or immobilization (Ebert *et al*., 2010). There is also evidence that ATF4 regulates obesity, energy expenditure, and glucose homeostasis, in that ATF4-null mice are lean and mildly hypoglycemic and resist obesity induced by aging or high energy diets (Yoshizawa *et al*., 2009; Wang *et al*., 2010; Miller *et al*., 2011). Effects on lipid metabolism are mediated through alterations of PGC1a in white and brown adipose tissue (Wang *et al*., 2013). Furthermore, ATF4-deficient mice show reductions in hypertriglyceridemia induced by high fructose diets (Zhu *et al*., 2013) and increased insulin sensitivity (Zhang *et al*., 2013), suggesting that ATF4 may act as a modulator of lipid metabolism in the liver.

The extent to which these diverse ATF4-modulated pathways might be involved in control of aging rate, longevity, and the development of neoplastic diseases, which are the most typical cause of death in mice, is at this point a matter for speculation and new experimentation. Construction of mice in which ATF4, or one or more of its immediate effectors, has been elevated or interrupted in specific cell types, or in which ATF4 pathways can be altered at specific postnatal time points, will also help to clarify the potential role played by ATF4 in postponement of disease and debility in aging animals. Evaluation of polymorphisms that modulate ATF4 level, or expression of a suite of ATF4-regulated mRNA, might also contribute to understanding of the genetic basis of specific disease of aging, or the relationship of genotype to overall health and longevity. Studies of ATF4 and its mediators in cells derived from long-lived and short-lived species could also produce insights into the possible role of the ATF4 system in evolutionary adaptation to niches that support slower aging and extended lifespan.

## Methods

### Mice and treatments

Acarbose: UM-HET3 mice, bred as the progeny of CB6F1 mothers and C3D2F1 fathers, were exposed to mouse chow containing acarbose at 1000 parts per million (ppm, equivalent to milligrams of drug per kilogram of chow) at 4 months of age and euthanized at 12 months of age. Mice in this population were also used for evaluation of hormonal and metabolic endpoints reported in (Harrison *et al*., 2013). Rapamycin: UM-HET3 mice were given food containing encapsulated rapamycin (Harrison *et al*., 2009) at 14 ppm from age 9 months and euthanized at 22 months of age. Calorie-restricted UM-HET3 mice (CR) were restricted to 80% of *ad libitum* food consumption from 6 to 8 weeks of age and to 60% of *ad libitum* consumption from 10 weeks of age until sacrifice at 12 months; metabolic and gene expression data from these mice were reported in (Miller *et al*., 2014). Mice fed with control mouse chow were used as controls for each of these three test groups; these control mice were born from the same breeding population, housed contemporaneously, and euthanized in parallel with the corresponding test group. CL12 mice were produced as described (Sun *et al*., 2009): experimental animals of the UM-HET3 stock were housed in groups of 12 newborns per foster mother from the first day of life until weaning at age 3 weeks, and then given free access to food after weaning. Controls for the CL12 mice were foster nursed in groups of 8 newborns per mother, and given free access to food after weaning. CL12 and their controls were euthanized at 6 months of age. Except for the acarbose group, mice were euthanized between 7 and 10 am and were not fasted prior to euthanasia. Mice in the acarbose group, and their controls, were fasted for four hr prior to sacrifice and killed between noon and 1 pm. All animal procedures were as approved by the University Committee on Use and Care of Animals at the University of Michigan. The colony is evaluated for specific pathogen status every 3 months, and all such tests were negative during the period of this study.

Methods for sample preparation, immunoblotting, and quantitative real-time PCR are as described in (Li & Miller, 2014).

### Statistics and graphics

Bar graphics show mean values for N = 6 individual mice of each sex and treatment group, and error bars indicate standard errors of the mean. Protein levels are expressed in each panel relative to the mean level of protein or mRNA in control mice of the same sex. mRNA values are expressed relative to the levels in control male mice.
